# Postoperative Outcomes of Transaxillary First Rib Resection with Anterior Scalenotomy for Thoracic Outlet Syndrome: An Ambispective Multimodal Cohort Study

**DOI:** 10.3390/medicina62040735

**Published:** 2026-04-12

**Authors:** Thrasyvoulos Michos, Anastasia Roumpaki, Emmanouil I. Kapetanakis, Petros Michos, Ioannis Gakidis, Christos Chantziantoniou, Aikaterini Kotroni, Ioanna Vlachou, Asterios Kanakis, Vicenzo Castilletti, Chara Tzavara, George Babis, Periklis Tomos, Spiros Pneumaticos

**Affiliations:** 1 Department of Thoracic Surgery, “Attikon” University Hospital, National and Kapodistrian University of Athens, 12 461 Athens, Greece; periklistomos@hotmail.com; 2 Liver Outpatient Unit, 3rd Internal Medicine Department, Evangelismos General Hospital, 106 76 Athens, Greece; ana_roumpaki@yahoo.com; 3 Department of General Thoracic Surgery, General Hospital of Attica “Κ.A.Τ”, 145 61 Athens, Greece; pmichos@yahoo.gr (P.M.); gakidis1958@gmail.com (I.G.); chatziantoniouc@gmail.com (C.C.); 4 Department of Physical Rehabilitation Medicine, General Hospital of Attica “Κ.A.Τ”, 145 61 Athens, Greece; katekot@otenet.gr (A.K.); asterioskanakis1@gmail.com (A.K.); 5 Radiology Department, General Hospital of Attica “Κ.A.Τ”, 145 61 Athens, Greece; ioannaareti@yahoo.gr; 6 Department of General Thoracic Surgery, 401 Military Hospital of Athens, 115 25 Athens, Greece; castilletiv@yahoo.gr; 7 Center of Health Services Research, Department of Hygiene, Epidemiology and Medical Statistics Medical School, National and Kapodistrian University of Athens, 11 527 Athens, Greece; htzavara@med.uoa.gr; 8 2nd Department of Orthopaedics, Konstantopouleio Hospital, National and Kapodistrian University of Athens, 10 559 Athens, Greece; george.babis@gmail.com; 9 3rd Department of Orthopaedics, General Hospital of Attica “Κ.A.Τ”, National and Kapodistrian University of Athens, 145 61 Athens, Greece; spirospneumaticos@gmail.com

**Keywords:** thoracic outlet syndrome, first rib resection, transaxillary approach, surgical outcomes, multimodal assessment

## Abstract

*Background and Objectives*: This study aimed to evaluate postoperative outcomes following transaxillary first rib resection with concomitant anterior scalenotomy (Roos procedure) for Thoracic Outlet Syndrome, using an ambispective design with a standardized two-year multimodal follow-up in a prospectively observed subgroup. *Materials and Methods*: This ambispective observational cohort study included 32 patients (87.5% women; mean age, 33.8 years) who underwent transaxillary first rib resection with anterior scalenotomy for Thoracic Outlet Syndrome. Of these, seven patients comprised the retrospective cohort, having undergone surgery between 2017 and 2019, while the remaining 25 patients were enrolled prospectively and underwent surgery from 2020 onwards. Patients were classified as having neurogenic, vascular (arterial or venous), or mixed Thoracic Outlet Syndrome. Retrospective data were obtained from medical records, while prospectively treated patients were followed according to a predefined postoperative protocol. Longitudinal changes in clinical outcomes were analyzed using mixed linear and logistic regression models. *Results*: All analyzed symptoms improved after surgery (*p* < 0.05), with a significant reduction in upper limb edema over time (OR = 0.44, *p* = 0.002). The prevalence of positive provocative tests decreased notably across all maneuvers postoperatively. Pathological color duplex ultrasound findings of the upper limb vessels resolved almost completely during follow-up. Patient-reported outcome measures (CBSQ, DASH, and BPI) demonstrated meaningful postoperative improvement with sustained benefits over time. Electrophysiological evaluation revealed notable improvement in median sensory and motor nerve conduction parameters. *Conclusions*: Transaxillary first rib resection with anterior scalenotomy appears to improve clinical, functional, and objective outcomes in patients with Thoracic Outlet Syndrome; however, findings should be interpreted with caution due to the ambispective design, small sample size, and cohort heterogeneity, and require confirmation in larger prospective studies.

## 1. Introduction

Thoracic outlet syndrome (TOS) encompasses a group of disorders resulting from compression of the neurovascular structures traversing the thoracic outlet and is classically categorized into neurogenic, venous, and arterial subtypes. Owing to its heterogeneous clinical presentation and the absence of universally accepted diagnostic criteria, TOS continues to pose significant diagnostic and therapeutic challenges despite advances in imaging and neurophysiological assessment [1].

In patients who remain symptomatic despite adequate conservative management, surgical decompression represents an established therapeutic option. Transaxillary first rib resection with concomitant anterior scalenotomy, commonly referred to as the Roos procedure, aims to relieve neurovascular compression by enlarging the thoracic outlet and eliminating contributing anatomical constraints [2]. Previous studies have reported generally favorable postoperative outcomes following this approach, with significant improvement in pain, upper-limb function, and quality of life in a substantial proportion of patients [3].

Mid- and long-term follow-up studies evaluating outcomes after first rib resection have demonstrated sustained functional improvement when assessed using validated patient-reported outcome measures, such as the Quick Disabilities of the Arm, Shoulder and Hand (QuickDASH) questionnaire [4]. Nevertheless, a subset of patients continues to experience residual or recurrent symptoms, underscoring the importance of systematic postoperative follow-up rather than reliance on short-term clinical improvement alone [4,5]. Despite the overall positive results reported in the literature, considerable heterogeneity exists with regard to follow-up duration, outcome measures, and methods of postoperative assessment. Previous reviews have emphasized the lack of standardized follow-up protocols and the wide variation in the use of subjective questionnaires versus objective diagnostic tools, including electrophysiological testing and vascular imaging [5]. This heterogeneity limits direct comparison between studies and hinders a comprehensive understanding of the durability and true extent of surgical benefit following decompression for TOS.

Accordingly, there remains a clear need for prospective studies employing predefined follow-up intervals and a multimodal assessment strategy integrating clinical examination, validated functional questionnaires, and objective diagnostic modalities. Standardized evaluation over a defined mid-term postoperative period may provide more robust evidence regarding both the effectiveness and durability of surgical treatment for Thoracic Outlet Syndrome.

The present study aims to evaluate postoperative outcomes following transaxillary first rib resection with concomitant anterior scalenotomy (Roos procedure) for TOS, incorporating a standardized two-year multimodal follow-up in a prospectively observed subgroup, while also including the postoperative course of patients who underwent the same procedure between 2017 and 2019, analyzed retrospectively.

## 2. Materials and Methods

### 2.1. Ethics Statement

The study was approved by the Scientific Review Board of the General Hospital of Attika “K.A.T.” (approval No. 48939; 30 October 2020). In addition, approval was obtained from the Bioethics and Ethics Committee of the Medical School of the National and Kapodistrian University of Athens. All participants provided written informed consent prior to study participation.

### 2.2. Study Design, Inclusion and Exclusion Criteria

**Figure 1:**
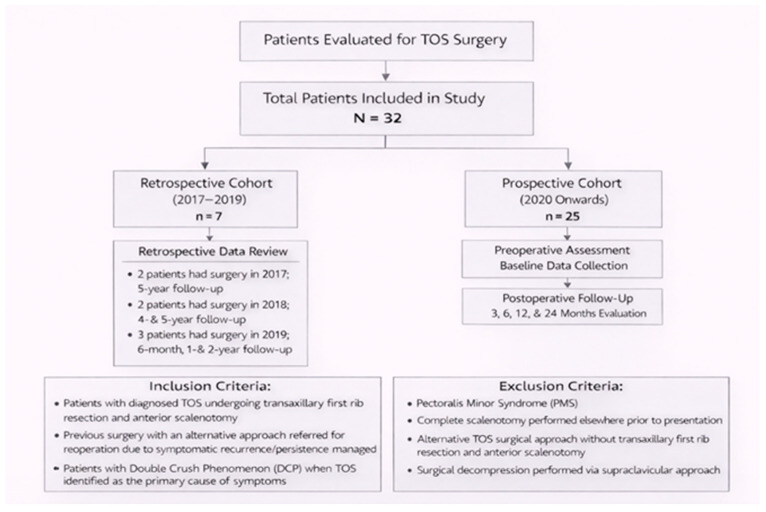
Flow diagram of patient inclusion, cohort allocation (retrospective and prospective), and postoperative follow-up schedule.

This study was designed as an observational cohort with both retrospective and prospective components. The study cohort consisted of consecutive patients who met the inclusion criteria and underwent surgical treatment during the study period. The retrospective arm comprised seven patients who underwent transaxillary first rib resection with concomitant anterior scalenotomy (Roos procedure) for TOS between 2017 and 2019, whose medical records were systematically reviewed. Specifically, two patients underwent surgery in 2017 and completed imaging and laboratory follow-up at 5 years postoperatively; two patients underwent surgery in 2018 and were evaluated at 4 and 5 years, whereas the remaining three patients underwent surgery in 2019 and were assessed at 6 months, 1 year, and 2 years postoperatively, in accordance with the available follow-up data (Figure 1). All patients in the retrospective cohort underwent standardized clinical re-evaluation, including detailed physical examination and validated clinical tests, and completed the same patient-reported outcome measures at predefined time points, aligned with those of the prospective cohort. Questionnaire administration and clinical assessments were performed in person during scheduled follow-up visits. At the time of surgical intervention, a standardized postoperative follow-up protocol incorporating electromyography (EMG) and upper limb duplex ultrasonography had not yet been established; consequently, comprehensive postoperative assessment with these modalities was not uniformly available across all retrospective cases.

The prospective arm included 25 patients who underwent surgical treatment between October 2020 and June 2025 and were followed according to a predefined postoperative protocol. All procedures were performed by four thoracic surgeons within our department, each with dedicated expertise in the Roos procedure.

Patients were classified according to TOS subtype as neurogenic, vascular, or mixed. Vascular TOS was further subdivided into arterial and venous subtypes based on the primary vascular structure involved. In addition, patients were stratified according to the interval between surgery and final postoperative evaluation (>10 years, 5–10 years, and <5 years).

The study included patients diagnosed with TOS who underwent surgical treatment via a transaxillary approach with first rib resection and concomitant anterior scalenotomy. In addition, patients who had previously been treated elsewhere using a different surgical approach and subsequently required reoperation due to symptom recurrence or persistence were also included, provided they were managed at the Thoracic Surgery Department of “K.A.T.” Hospital as a referral center. In these patients, although division of the anterior scalene muscle had been documented in prior operative reports, intraoperative findings during the revision procedure demonstrated that the muscle remained intact, and anterior scalenotomy was therefore performed during the second intervention.

Patients diagnosed with Double Crush Phenomenon (DCP) were eligible for inclusion when TOS was identified as the predominant cause of clinical symptomatology. In such cases, cervical magnetic resonance imaging was reviewed, and preoperative evaluation by a neurosurgical team was undertaken to exclude cervical spine pathology as the primary source of symptoms. Additionally, an orthopedic evaluation was performed to rule out ulnar neuropathy or carpal tunnel syndrome. Following completion of these assessments and confirmation of clinical findings consistent with TOS, patients were considered appropriate candidates for surgical treatment.

Patients with Pectoralis Minor Syndrome (PMS) were not included in the study. Exclusion criteria comprised patients in whom a complete scalenotomy had already been performed at another institution, as well as those treated surgically using alternative approaches without transaxillary first rib resection and anterior scalenotomy. Patients who underwent surgical decompression via a supraclavicular approach at the Thoracic Surgery Department of “K.A.T” Hospital were also excluded from the analysis.

### 2.3. Data Collection and Preoperative Assessment

Demographic and epidemiological data were collected for all patients, including age, sex, athletic activity, and history of trauma involving the affected upper limb or cervical spine. Particular emphasis was placed on the duration of symptoms prior to surgical intervention, as well as on the use of preoperative physiotherapy and the total number of physiotherapy sessions completed.

Preoperative diagnostic evaluation included electromyography, chest and cervical spine radiographs, computed tomography or magnetic resonance imaging of the cervical spine, and color duplex ultrasound examination of the upper limb vessels. When clinically indicated, arterial or venous angiography was additionally performed.

### 2.4. Outcome Measures

Patient-reported outcome measures were used to evaluate postoperative upper-limb function, pain severity, and their impact on daily activities and quality of life. Functional status of the operated upper extremity was assessed using the validated Greek version of the Quick Disabilities of the Arm, Shoulder and Hand (QuickDASH) questionnaire. Pain intensity and pain-related interference with daily activities were evaluated using the Greek version of the Brief Pain Inventory (BPI). In addition, cervical and brachial symptoms were assessed using the Cervical Brachial Symptom Questionnaire (CBSQ).

Patient-reported outcomes were complemented by standardized clinical examination and the use of provocative tests to assess symptom reproduction and functional limitation.

### 2.5. Postoperative Evaluation and Follow-Up

Final postoperative evaluation included comprehensive medical history taking and physical examination, vascular assessment of the thoracic outlet using duplex ultrasound (Philips Affiniti 30 Ultrasound System, Philips Healthcare, Amsterdam, Netherlands), electrophysiological testing, and chest radiography. Patients were also asked to complete postoperative questionnaires assessing symptom progression and functional outcomes.

The prospective arm of the study included patients who underwent surgical treatment from October 2020 through June 2025. These patients were followed according to a predefined postoperative protocol with scheduled evaluations at 3, 6, 12, and 24 months after surgery. Each follow-up visit included clinical examination, vascular imaging, electrophysiological assessment, and completion of patient-reported outcome questionnaires.

The retrospective arm comprised seven patients who underwent surgery between 2017 and 2019, whose medical records were systematically reviewed. Specifically, two patients (2017) had 5-year follow-up; two patients (2018) had follow-up at 4 and 5 years; and three patients (2019) were evaluated at 6 months, 1 year, and 2 years postoperatively 

(Figure 1). All patients underwent standardized clinical re-evaluation, including physical examination, validated clinical tests, and completion of the same patient-reported outcome questionnaires at predefined time points consistent with the prospective cohort. However, comprehensive postoperative assessment with electromyography (EMG) and vascular duplex ultrasonography was not available in all retrospective cases.

### 2.6. Statistical Analysis

Quantitative variables were summarized as mean values with standard deviation or as median values with interquartile range, as appropriate, while categorical variables were presented as absolute and relative frequencies. Normality of continuous variables was assessed using the Kolmogorov–Smirnov test. No imputation was performed for missing data.

Longitudinal changes over time were evaluated using mixed linear or mixed logistic regression models to account for repeated measurements obtained at different time points within the same patient and to allow for missing observations. Adjusted regression coefficients (β) with corresponding 95% confidence intervals (95% CI) were derived from mixed linear models, while odds ratios with 95% confidence intervals were obtained from mixed logistic regression analyses. Logarithmic transformation of dependent variables was applied when normality assumptions were not met. Nonlinear associations were explored using spline regression with a single knot at 3 months. Interaction terms were included to assess effect modification by TOS subtype and symptom duration.

All statistical tests were two-tailed, with statistical significance set at *p* < 0.05. Statistical analyses were performed using STATA statistical software (version 15.0). The number of observations included in each analysis is reported accordingly. While no missing data were present for the primary longitudinal clinical and patient-reported outcomes, certain analyses (e.g., electrophysiological and vascular imaging assessments) were based on subsets of patients with available data, particularly within the retrospective cohort.

## 3. Results

Data from 32 patients (87.5% women) were collected and analyzed, with a mean age of 33.8 years (SD = 11.5). Baseline demographic and clinical characteristics are summarized in Table 1. Mixed-type TOS accounted for 43.8% of the study population, while 65.6% of patients reported symptom duration of less than five years. Preoperative physiotherapy had been undertaken by 46.9% of patients, and 59.4% reported regular physical activity. The right upper limb was affected in 59.4% of cases. Double Crush Syndrome was identified in 25.0% of patients, and 65.6% presented with concomitant cervical pathology.

Intraoperatively, pneumothorax occurred in 12.5% of patients, whereas no cases of hemothorax were observed. No instances of phrenic nerve injury, hemidiaphragm paresis, or postoperative surgical site infection were recorded. One patient (3.1%) experienced intraoperative long thoracic nerve injury resulting in winged scapula, and six patients (18.8%) sustained musculocutaneous nerve injury associated with decreased sensation over the medial aspect of the arm.

Patients’ symptoms and clinical test results throughout the follow-up period are presented in Table 2. Preoperatively, the most prevalent symptom was paresthesia and numbness (87.5%), followed by arm pain, forearm pain, and loss of grip strength (65.6% each). Postoperatively, the most frequently reported symptoms were paresthesia and numbness, as well as shoulder girdle pain. Upper limb edema was present in 37.5% of patients preoperatively, decreased to 9.4% at two years postoperatively, and was absent during the intervening follow-up assessments.

Preoperatively, the proportion of positive provocative tests ranged from 28.1% (pectoralis minor compression test) to 96.9% (hyperabduction maneuver). Additionally, 68.8% of patients demonstrated pathological duplex ultrasound findings of the upper limb vessels. Postoperatively, the frequency of positive provocative tests ranged from 3.1% (pectoralis minor compression test at one year) to 34.4% (hyperabduction maneuver at three months). No patient exhibited pathological duplex ultrasound findings at three months, one year, or two years of follow-up, while one patient (3.1%) demonstrated abnormal duplex ultrasound results at six months.

Mixed logistic regression analysis demonstrated statistically significant changes over time for all analyzed symptoms (Table 3). Specifically, upper limb edema decreased significantly during follow-up (OR = 0.44, *p* = 0.002). Postoperatively, notable reductions were observed in all other analyzed symptoms compared with the preoperative period (OR range: 0.0001–0.04; *p* < 0.05). During the preoperative period, only hand pain showed a significant increase over time (OR = 21.06, *p* = 0.015), whereas no significant changes were detected for the remaining symptoms.

The likelihood of a positive hyperabduction maneuver (Wrights test), costoclavicular maneuver (Eden test—Military brace maneuver), EAST test (Elevated Arm Stress Test), Adson test, scalene triangle compression test and pectoralis minor compression test was significantly reduced postoperatively compared with the preoperative period (OR range: 0.0001–0.138; *p* < 0.05). During postoperative follow-up, the probability of positive results in these tests did not vary significantly (*p* > 0.05). The probability of pathological color duplex ultrasound findings demonstrated a decreasing trend after surgery (OR = 0.001; *p* = 0.066) and remained stable throughout the postoperative period (OR = 0.64; *p* = 0.654).

**Figure 2:**
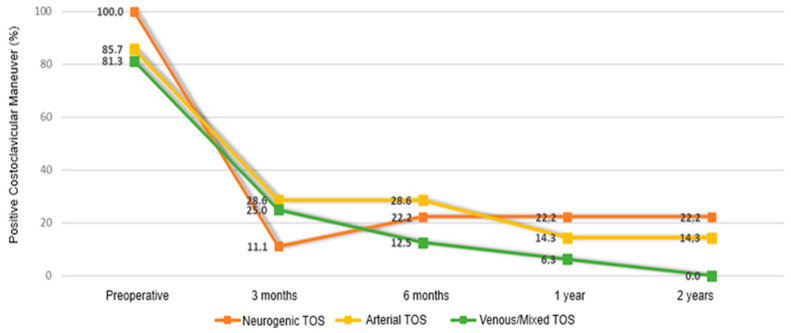
Changes in positive Costoclavicular Maneuver throughout follow-up, by TOS type.

**Figure 3:**
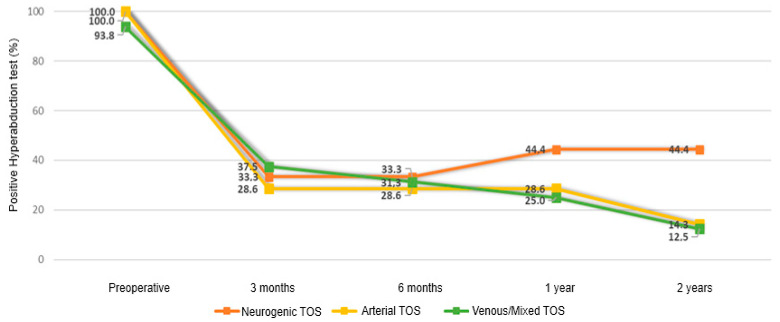
Changes in positive Hyperabduction Maneuver throughout follow-up, by TOS type.

Temporal changes were not significantly associated with symptom duration or TOS subtype for most outcomes (all interaction terms *p* > 0.05), with the exception of the hyperabduction maneuver and costoclavicular maneuver. Specifically, a significant reduction in the probability of a positive costoclavicular maneuver was observed across all TOS subtypes during follow-up (OR = 0.40, 95% CI: 0.21–0.75; *p* = 0.004); however, this reduction was significantly less pronounced in patients with mixed or venous TOS compared with those with neurogenic TOS (interaction term: OR = 0.25, 95% CI: 0.07–0.98; *p* = 0.046; Figure 2). Similarly, a meaningful reduction in the probability of a positive hyperabduction maneuver was observed across all TOS subtypes (OR = 0.50, 95% CI: 0.28–0.92; *p* = 0.026; Figure 3), with a smaller reduction in mixed or venous TOS compared with neurogenic TOS (interaction term: OR = 0.34, 95% CI: 0.12–0.97; *p* = 0.044; Figure 3).

Descriptive statistics for the CBSQ, DASH, and BPI scales are presented in Table 4. Preoperatively, the mean CBSQ score was 76.72 (SD = 23.75), the mean DASH score was 62.76 (SD = 21.23), the mean BPI severity score was 5.83 (SD = 3.11), and the mean BPI interference score was 5.70 (SD = 2.14). All scales demonstrated significant postoperative improvement compared with preoperative values (OR range: −0.78 to −0.38; *p* < 0.05; Table 5). During postoperative follow-up, no significant temporal changes were observed in CBSQ, DASH, or BPI interference scores (*p* > 0.05), whereas the BPI severity score continued to decline significantly over time (OR = −0.08; *p* = 0.044). These changes were not significantly associated with symptom duration or TOS subtype (all interaction terms *p* > 0.05).

Descriptive measures of sensory and motor nerve conduction are presented in Table 6. Ulnar sensory nerve values did not change significantly during follow-up (β = 0.017, 95% CI: −0.027 to 0.061; *p* = 0.464). Median sensory nerve values increased notably postoperatively compared with preoperative measurements (β = 0.27, 95% CI: 0.07–0.48; *p* = 0.007) but did not change significantly throughout the postoperative period (β = −0.05, 95% CI: −0.12 to 0.02; *p* = 0.169). A notable increase over time was observed in median motor nerve values (β = 0.06, 95% CI: 0.02–0.11; *p* = 0.003) and in ulnar motor nerve values (β = 0.05, 95% CI: 0.02–0.08; *p* = 0.001). Ulnar motor nerve measurements at Erb’s point demonstrated an increasing trend over time that did not reach statistical significance (β = 0.07, 95% CI: −0.01 to 0.14; *p* = 0.068). None of these temporal changes were significantly associated with symptom duration or TOS subtype (all interaction terms *p* > 0.05).

## 4. Discussion

The present ambispective cohort study demonstrated meaningful improvement in clinical symptoms, functional outcomes, electrophysiological parameters, and provocative test results following transaxillary first rib resection with concomitant anterior scalenotomy for Thoracic Outlet Syndrome. These findings further support surgical decompression as an effective therapeutic option for patients with persistent symptoms despite adequate conservative management, in line with previous observational studies and systematic reviews [3,4,5,6,7,8]. Importantly, the observed improvement was documented using both patient-reported outcome measures and objective diagnostic modalities, thereby supporting the use of a multimodal approach in the evaluation of postoperative recovery.

Postoperative functional improvement, as reflected by significant reductions in QuickDASH, CBSQ, and BPI scores, aligns with prior studies evaluating patient-reported outcomes following first rib resection. Long-term series have consistently demonstrated significant improvement in pain, upper-limb function, and quality of life following surgical decompression, although residual disability compared with the general population may persist in a subset of patients [4,9,10,11]. Meta-analytic evidence further supports favorable functional outcomes across Thoracic Outlet Syndrome subtypes after surgical treatment [5,8]. In the present study, functional and pain-related outcomes improved substantially after surgery and subsequently demonstrated a tendency toward stabilization, suggesting that the main therapeutic benefit is achieved during the early postoperative period, while sustained improvement reflects the long-term effectiveness of decompression.

Of particular clinical relevance, pathological findings on color duplex ultrasound examination of the upper limb vessels resolved almost completely during postoperative follow-up. This observation supports the effectiveness of surgical vascular decompression and underscores the role of color duplex ultrasound as a valuable objective tool not only in the initial diagnostic workup but also in postoperative surveillance and longitudinal assessment of recovery.

The long-term durability of surgical outcomes remains a critical consideration in the management of Thoracic Outlet Syndrome. Cohort studies with follow-up exceeding ten years have demonstrated sustained symptom relief and functional improvement following first rib resection, particularly in neurogenic Thoracic Outlet Syndrome [12,13,14]. Earlier large surgical series similarly reported durable clinical benefits in the majority of patients at extended follow-up [15,16]. The stability of functional scores and symptom improvement observed over time in the present cohort is consistent with these long-term data and further supports the lasting effectiveness of surgical decompression.

Differences in surgical outcomes among TOS subtypes have been previously described. Several studies suggest that arterial and venous forms may achieve more predictable symptomatic relief compared with purely neurogenic TOS, likely reflecting differences in underlying pathophysiology and objective compressive lesions [5,17,18,19]. In the present cohort, however, overall clinical response was largely comparable across subtypes, with limited subtype-specific differences observed primarily in selected provocative maneuvers, such as the hyperabduction and costoclavicular tests. These findings should be interpreted as exploratory given the limited sample size and suggest that, despite underlying pathophysiological differences, surgical decompression may result in comparable functional benefits, while variations in specific clinical tests likely reflect differing compression mechanisms rather than meaningful differences in overall outcome.

The relationship between preoperative physiotherapy and postoperative recovery also merits consideration. As most patients were referred to our department after unsuccessful physiotherapy, no standardized preoperative protocol could be defined. In general, physiotherapy includes stretching of the scalene and pectoral muscles, scapular mobility exercises, postural and diaphragmatic training, and progressive strengthening. The overall aim is to reduce neurovascular compression by improving muscular balance and thoracic outlet biomechanics [20,21]. Although preoperative physiotherapy did not appear to influence final functional outcomes, it was associated with differences in the timing of postoperative improvement. This observation indicates that, in patients with persistent symptoms, surgical decompression appears to be the primary determinant of recovery, independent of prior conservative treatment strategies.

The low complication rate observed in this study is consistent with contemporary surgical series reporting acceptable perioperative morbidity following transaxillary first rib resection when performed in experienced centers [22,23,24,25,26,27]. Pneumothorax and transient nerve injuries are among the most frequently reported complications, whereas permanent neurological deficits and major vascular injuries remain uncommon [23,25,28]. Additionally, the observed improvement in electrophysiological parameters without a corresponding increase in pathological findings on physiatric assessment is consistent with recovery of neural conduction without evidence of clinically significant muscle weakness, further supporting the safety profile of the procedure.

Despite overall favorable outcomes, persistent or recurrent symptoms continue to be reported in a subset of patients following decompression. Previous studies have emphasized the multifactorial nature of TOS and the influence of coexisting cervical spine pathology, double-crush phenomena, and chronic pain syndromes on postoperative recovery [4,11,15,29,30,31]. The present findings reinforce the importance of careful patient selection and realistic preoperative counseling regarding expected outcomes. In patients with persistent postoperative symptoms, particularly following long-standing preoperative symptomatology, adjunctive physiotherapy should be considered. Early management focuses on pain control, preservation of range of motion, and prevention of muscle spasm, followed by gradual strengthening after approximately 8 weeks and progressive return to function. A structured rehabilitation approach is essential to minimize the risk of symptom recurrence, with full recovery typically achieved within 9–12 months following surgical decompression [20].

One of the major strengths of the present study is the use of a standardized multimodal follow-up protocol integrating patient-reported outcome measures, clinical examination, provocative testing, vascular imaging, and electrophysiological assessment. Prior reviews and consensus statements have highlighted the heterogeneity of diagnostic criteria, outcome measures, and follow-up strategies across Thoracic Outlet Syndrome studies [1,5,31,32]. By employing a comprehensive longitudinal assessment framework, the present study supports the need for standardized reporting and structured follow-up in future research.

Several limitations of the present study should be acknowledged. First, the relatively small sample size and single-center design may limit the generalizability of the findings. In addition, the ambispective design represents an inherent limitation, as data collection was not fully uniform between the retrospective and prospective components, potentially introducing heterogeneity in measurement procedures. Given the relatively small sample size, subgroup and interaction analyses should be considered exploratory in nature. Therefore, any comparisons between TOS subtypes or other stratified analyses should be interpreted with caution and not as definitive evidence of differential treatment effects. To mitigate these concerns, advanced statistical modeling techniques were applied to appropriately handle potential sources of bias and to enable the derivation of statistically robust estimates. Nonetheless, the implementation of a standardized multimodal follow-up protocol, combined with longitudinal outcome assessment, enhances the internal validity of the study findings.

It should be noted that the study included 32 patients, each with repeated measurements at five predefined time points (one preoperative and four postoperative assessments), yielding a total of 160 observations for the longitudinal analyses. Importantly, there were no missing data across follow-up time points, allowing inclusion of all available observations in the statistical models. Linear mixed-effects models were selected as the primary analytical approach, given their suitability for longitudinal data with repeated measures, even in relatively small samples. These models appropriately account for within-subject correlation and allow efficient use of all available data points. However, the limited sample size may reduce the robustness of subgroup and interaction analyses, and these findings should therefore be interpreted with caution. Despite this limitation, the main longitudinal trends in outcomes were consistent and statistically significant, supporting the overall conclusions of postoperative improvement. Future prospective multicenter studies with larger cohorts and longer follow-up are warranted to further refine patient selection criteria and to support standardized outcome reporting following surgical treatment for Thoracic Outlet Syndrome.

## 5. Conclusions

Transaxillary first rib resection with anterior scalenotomy appears to improve clinical, functional, and objective outcomes in patients with Thoracic Outlet Syndrome. Nevertheless, the ambispective design, limited sample size, and cohort heterogeneity warrant cautious interpretation. Further prospective studies with standardized assessment protocols are required to validate these findings.

## Figures and Tables

**Table 1:** Sample characteristics.

*n* = 32		*n*	%
Sex			
	Men	4	12.5
	Women	28	87.5
T.O.S type			
	Neurogenic	9	28.1
	Arterial	7	21.9
	Mixed	14	43.8
	Venous	2	6.3
Duration of symptoms			
	<5 years	21	65.6
	5–10 years	4	12.5
	>10 years	7	21.9
Physiotherapy preoperatively		15	46.9
Physical activities		19	59.4
Side			
	Right	19	59.4
	Left	13	40.6
Double Crush Syndrome (DCS)		8	25.0
Revision Transaxillary First Rib Resection		0	0.0
Revision Scalenotomy		6	18.8
Cervical Rib		1	3.1
Cervical Spine Pathology		21	65.6
Upper Extremity and Cervical Spine Injuries		9	28.1
		Mean (SD)	Median (IQR)
Age (years)		33.8 (11.5)	36 (25–42.5)
Duration of symptoms (years)		4.9 (5)	3 (1–7)

**Table 2:** Patients’ symptoms and clinical maneuver test results throughout the follow-up period.

	Preoperative		3 Months Postoperative		6 Months Postoperative		1 Year Postoperative		2 Year Postoperative	
	*n*	%	*n*	%	*n*	%	*n*	%	*n*	%
Paresthesia and numbness	28	87.5	18	56.3	17	53.1	21	65.6	19	59.4
Brachial pain	21	65.6	1	3.1	3	9.4	3	9.4	3	9.4
Forearm pain	21	65.6	0	0.0	2	6.3	0	0.0	2	6.3
Loss of pulses	21	65.6	1	3.1	1	3.1	1	3.1	1	3.1
Hand pain	19	59.4	1	3.1	1	3.1	4	12.5	4	12.5
Shoulder girdle pain	18	56.3	4	12.5	5	15.6	6	18.8	7	21.9
Skin discoloration—Pallor	16	50.0	1	3.1	2	6.3	1	3.1	2	6.3
Upper extremity weakness	15	46.9	3	9.4	2	6.3	3	9.4	3	9.4
Cervical spine pain	12	37.5	1	3.1	1	3.1	3	9.4	0	0.0
Alteration in limb temperature	12	37.5	1	3.1	1	3.1	0	0.0	2	6.3
Chest wall pain	10	31.3	0	0.0	1	3.1	0	0.0	2	6.3
Ischemia—Raynaud’s phenomenon	3	9.4	0	0.0	0	0.0	0	0.0	0	0.0
Muscle atrophy	2	6.3	0	0.0	0	0.0	0	0.0	0	0.0
Superficial venous distension	2	6.3	1	3.1	0	0.0	0	0.0	0	0.0
Headaches	1	3.1	0	0.0	0	0.0	0	0.0	0	0.0
Hyperabduction Maneuver	31	96.9	11	34.4	10	31.3	10	31.3	7	21.9
Positive Costoclavicular Maneuver	28	87.5	7	21.9	6	18.8	4	12.5	3	9.4
Positive EAST	27	84.4	9	28.1	6	18.8	4	12.5	5	15.6
Positive Adson’s Maneuver	26	81.3	7	21.9	8	25.0	7	21.9	7	21.9
Positive Scalene Triangle Compression Test	22	68.8	5	15.6	7	21.9	3	9.4	5	15.6
Abnormal color duplex ultrasound findings of the upper limb vessels	22	68.8	0	0.0	1	3.1	0	0.0	0	0.0
Positive Pectoralis Minor Compression Test	9	28.1	3	9.4	3	9.4	1	3.1	3	9.4

**Table 3:** Results from mixed regression models for patients’ symptoms and clinical maneuver tests.

	Postoperative vs. Preoperative		During Postoperative Period	
	OR (95% CI) +	*p*	OR (95% CI) +	*p*
Paresthesia and numbness	0.04 (0.006–0.24)	<0.001	1.21 (0.76–1.91)	0.420
Brachial pain	0.03 (0.01–0.12)	<0.001	1.30 (0.72–2.36)	0.381
Forearm pain	0.01 (0.001–0.100)	<0.001	1.55 (0.59–4.05)	0.374
Loss of pulses	0.0001 (0.000–0.0001)	<0.001	1.00 (0.03–33.23)	>0.999
Hand pain	0.0001 (0.000–0.0001)	<0.001	21.06 (1.81–245.24)	0.015
Shoulder girdle pain	0.02 (0.01–0.16)	<0.001	1.40 (0.0.83–2.37)	0.204
Skin discoloration—Pallor	0.005 (0.0003–0.10)	0.001	1.23 (0.50–2.99)	0.655
Upper extremity weakness	0.02 (0.003–0.17)	<0.001	1.07 (0.53–2.16)	0.857
Cervical spine pain	0.03 (0.003–0.29)	0.002	0.91 (0.38–2.16)	0.826
Alteration in limb temperature	0.03 (0.004–0.30)	0.002	1.23 (0.50–3.07)	0.650
Chest wall pain	0.003 (0.000–0.23)	0.009	2.58 (0.60–11.07)	0.202
Hyperabduction Maneuver	0.0001 (0.0003–0.03)	0.002	0.64 (0.36–1.13)	0.121
Positive Costoclavicular Maneuver	0.005 (0.0003–0.07)	<0.001	0.58 (0.33–1.05)	0.071
Positive EAST	0.0001 (0.000–0.08)	0.008	0.35 (0.12–1.05)	0.060
Positive Adson’s Maneuver	0.03 (0.0002–0.04)	<0.001	0.96 (0.54–1.69)	0.885
Positive Scalene Compression Test	0.01 (0.001–0.09)	<0.001	0.83 (0.46–1.52)	0.547
Abnormal color duplex ultrasound findings of the upper limb vessels.	0.001 (0.000–1.54)	0.066	0.64 (0.09–4.48)	0.654
Positive Pectoralis Minor Compression Test	0.138 (0.027–0.707)	0.018	0.89 (0.46–1.73)	0.738

+ Odds Ratio (95% Confidence Interval) for time effect.

**Table 4:** Descriptive measures for CBSQ, DASH and BPI scales.

	CBSQ		DASH		BPI Severity Score		BPI Interference Score	
	Mean (SD)	Median (IQR)	Mean (SD)	Median (IQR)	Mean (SD)	Median (IQR)	Mean (SD)	Median (IQR)
Preoperative	76.72 (23.75)	80 (64–92)	62.76 (21.23)	64.25 (43.71–80.5)	5.83 (3.11)	5.75 (4.23–7)	5.7 (2.14)	5.88 (4.79–7.16)
3 months postoperative	42.2 (26.67)	36 (22.5–64.5)	37.79 (17.33)	38.1 (27.9–48.78)	3.88 (2.15)	3.88 (2.49–5)	3.69 (2.41)	3.24 (1.93–5.07)
6 months postoperative	44.5 (29.02)	38.5 (25–77)	38.51 (23.81)	40.5 (17.7–50)	3.89 (2.39)	3.47 (1.75–6)	3.73 (2.84)	2.7 (1.5–5.6)
1 year postoperative	41.3 (26.93)	34 (20–68)	34.53 (18.77)	34.25 (18.9–46)	3.14 (2.18)	2.74 (1.15–5)	3.26 (2.45)	2.22 (1.71–5.75)
2 years postoperative	36.39 (26.34)	34 (13–61.5)	33.65 (20.48)	31.3 (16.9–47.5)	3.22 (2.6)	2.13 (1.25–5.38)	3.46 (2.88)	2.43 (1.15–5.88)

**Table 5:** Mixed linear regression analysis results for CBSQ, DASH and BPI scales.

	Postoperative vs. Preoperative		During Postoperative Period	
	β (95% CI) +	*p*	β (95% CI) +	*p*
CBSQ	−0.78 (−1.05–−0.50)	<0.001	−0.07 (−0.17–0.03)	0.173
DASH	−0.71 (−1.03–−0.40)	<0.001	−0.03 (−0.14–0.08)	0.574
BPI severity score	−0.38 (−0.60–−0.15)	0.001	−0.08 (−0.16–−0.002)	0.044
BPI interference score	−0.45 (−0.66–−0.24)	<0.001	−0.05 (−0.13–0.02)	0.144

Note. The analysis was performed after having the dependent variables logarithmically transformed due to the asymmetry of their distribution. + Regression coefficient (95% Confidence Interval) for time effect.

**Table 6:** Descriptive measures for sensory and motor nerves.

	Median Sensory Nerve (uv)		Ulnar Sensory Nerve (uv)		Median Motor Nerve (mv)		Ulnar Motor Nerve (mv)		Ulnar Motor Nerve Erb (mv)	
	Mean (SD)	Median (IQR)	Mean (SD)	Median (IQR)	Mean (SD)	Median (IQR)	Mean (SD)	Median (IQR)	Mean (SD)	Median (IQR)
Preoperative	51.23 (32.86)	47.5 (25.2–76.65)	41.52 (25.28)	35 (25–56.2)	7.96 (3.93)	6.8 (5.6–9.1)	8.23 (2.46)	8.2 (6.9–8.9)	3.8 (2.6)	3.4 (1.21–6.5)
3 months postoperative	65.06 (30.82)	51 (45.8–89.4)	35.42 (23.18)	31.2 (17.3–42.5)	8.08 (3.65)	8 (5.2–9.9)	8.18 (1.91)	8.2 (6.9–8.9)	3.91 (3.85)	3 (1.21–5.8)
6 months postoperative	59.52 (29.76)	44 (40.9–74)	38.96 (24.93)	36.05 (22–44.1)	9.71 (5.93)	8.35 (5–12.8)	9.95 (3.94)	9.1 (7.7–10.5)	3.42 (2.8)	3.6 (1.41–5.3)
1 year postoperative	62.33 (28.97)	71.9 (34–82.1)	39.39 (25.28)	34.5 (23–62.2)	12.93 (13.18)	7.8 (6.4–12)	12.63 (11.91)	9 (8–12.8)	3.82 (2.46)	3.65 (1.87–5.4)
2 years postoperative	53.84 (31.44)	50.05 (32.6–71.6)	44.28 (26.88)	42.1 (25.2–57.9)	11.21 (10.21)	8.6 (6–12)	10.96 (9.28)	9.5 (7–12.2)	5.84 (3.96)	5.85 (2.6–8.05)

Analyses based on available cases.

## Data Availability

Data access is strictly limited to the authors specifically as regulated by authority approvals and therefore not possible to share.

## Abbreviations

BPI	Brief Pain Inventory
CBSQ	Cervical Brachial Symptom Questionnaire
CI	Confidence Interval
CT	Computed Tomography
DASH	Disabilities of the Arm, Shoulder and Hand
DCP	Double Crush Phenomenon
EAST	Elevated Arm Stress Test
EMG	Electromyography
IQR	Interquartile Range
MRI	Magnetic Resonance Imaging
OR	Odds Ratio
PMS	Pectoralis Minor Syndrome
PROMs	Patient-Reported Outcome Measures
SD	Standard Deviation
TOS	Thoracic Outlet Syndrome
